# Detection and quantification of water-based aerosols using active open-path FTIR

**DOI:** 10.1038/srep25110

**Published:** 2016-04-28

**Authors:** Oz Kira, Raphael Linker, Yael Dubowski

**Affiliations:** 1 Faculty of Civil and Environmental Engineering, Technion-Israel Institute of Technology, Technion City, Haifa 3200003, Israel

## Abstract

Aerosols have a leading role in many eco-systems and knowledge of their properties is critical for many applications. This study suggests using active Open-Path Fourier Transform Infra-Red (OP-FTIR) spectroscopy for quantifying water droplets and solutes load in the atmosphere. The OP-FTIR was used to measure water droplets, with and without solutes, in a 20 m spray tunnel. Three sets of spraying experiments generated different hydrosols clouds: (1) tap water only, (2) aqueous ammonium sulfate (0.25–3.6%wt) and (3) aqueous ethylene glycol (0.47–2.38%wt). Experiment (1) yielded a linear relationship between the shift of the extinction spectrum baseline and the water load in the line-of-sight (LOS) (R^2^ = 0.984). Experiment (2) also yielded a linear relationship between the integrated extinction in the range of 880–1150 cm^−1^ and the ammonium sulfate load in the LOS (R^2^ = 0.972). For the semi-volatile ethylene glycol (experiment 3), present in the gas and condense phases, quantification was much more complex and two spectral approaches were developed: (1) according to the linear relationship from the first experiment (determination error of 8%), and (2) inverse modeling (determination error of 57%). This work demonstrates the potential of the OP-FTIR for detecting clouds of water-based aerosols and for quantifying water droplets and solutes at relatively low concentrations.

Airborne particles influence significantly on our health and environment^1^,^2^. Pollution due to airborne particles from vehicles, industry, and agriculture led numerous organizations to view the development of monitoring techniques for aerosols as a high priority topic. Environmental remote sensing platforms have shown great potential for assisting different monitoring projects from satellite measurements of water quality^3^,^4^,^5^ air pollution^6^,^7^, and dust^8^,^9^ to earth based devices which measures fugitive gases from the industry^10^. Still, one of the main challenges in the field of environmental monitoring remains the measurement of airborne particulate matter.

One of the ground-based devices that have great potential is the light detection and ranging (LIDAR) device, which has the ability to quantify aerosols and to determine their size distribution. In recent studies^11^,^12^, it was demonstrated that LIDAR ground measurements allowed the estimation of drift of particulate matter emitted during agricultural operations. Another technique that has shown great potential for monitoring aerosols is Open Path Fourier Transform Infra-Red (OP-FTIR) spectroscopy. While LIDAR operates in the UV-VIS-NIR range and usually has one laser operating at a specific wavelength, the OP-FTIR operates in the mid-IR range where many organic compounds present unique spectral “fingerprints”, adding potential for gaining chemical information of the aerosols. The OP-FTIR, which is the main focus of this study, has been successfully applied for measurement of gaseous compounds (e.g., fugitive gases from petrochemical tank^13^ and volcanic gas emissions^14^). A few studies have shown the potential of OP-FTIR for aerosols detection, e.g. measurements of water droplets^15^,^16^, and simulations of ammonium nitrate and ammonium sulfate^16^. Nevertheless, aerosols quantification was obtained only by combination of LIDAR and OPFTIR^17^,^18^. The current study intends to broaden the OP-FTIR capabilities and to allow quantification of hydrosols and of the solutes inside them.

When measuring gases in the mid-IR range (λ = 2–20 μm) the spectral signal is determined mostly by the gas absorption. On the other hand, when monitoring aerosols in the micrometer size range, as in the present study, the use of the generalized Mie scattering theory is essential^19^. When using active sensing (using an artificial IR source) the measured signal is in the form of an extinction spectrum. The connection between the measured signal and the particle properties is through the wavelength-dependent extinction cross section^20^ (*σ*_*e*_), which is calculated using coefficients developed on the basis of spherical wave equations^20^:


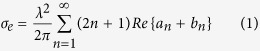


where Re{*a*_*n*_ + *b*_*n*_} is the real part of the coefficients *a*_*n*_ and *b*_*n*_, and λ is the incidence wavelength. The Mie coefficients for the scattered field *a*_*n*_ and *b*_*n*_ are obtained by the boundary conditions of the electric and magnetic fields at a spherical particle’s surface. The connection between the total extinction cross section (right-hand side of equation (2)) and the measured extinction spectrum (left-hand side of equation (2)) is^20^ given by:


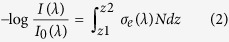


where *I(λ)* and *I*_*0*_*(λ)* are the radiation intensities sensed (with and without aerosols in the line-of-sight, respectively), *z*1 and *z*2 indicate the cloud’s boundaries, and *N* is the number density of the aerosols in the cloud. The extinction spectrum, which is the product of the OP-FTIR measurements, is dependent on the ratio of the radiant flux received by the sensor with and without aerosols in the line-of-sight.

In many cases the aerosols are composed of more than one component. The spectral measurements in these cases will include the signatures of all the present components and the analysis will demand the isolation of each contribution. The computation of the extinction cross section of aerosols depends on the refractive index of the particles. When dealing with internally mixed aerosols^21^, which are composed of two or more substances, the refractive index of the aerosols can be evaluated using linear mixing rules of the pure components. For binary solution/particle aerosols (the simplest case), the linear mixing rule^22^,^23^ is:


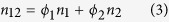


where *n*_i_ and *ϕ*_i_ are the refractive index and volume fraction of substance i.

Additionally, semi-volatile components in aerosols can evaporate, which adds more spectral signatures as the signals of the aerosols and the vapors often differ. Thus quantification of all components appears to be a very challenging task.

The objectives of the current study were to demonstrate the suitability of active OP-FTIR for quantifying water droplets load and to investigate the ability to detect and quantify solutes (volatile and non-volatile) in the droplets.

## Methods

Spraying experiments were conducted in a special spraying tunnel at the Technion-Israel Institute of Technology Campus, which included a custom spraying system based on common agricultural sprayers. The experiments were divided into three main types: 1) Tap water was sprayed at five different loads to obtain a calibration curve for water load in the line of sight (LOS). 2) Five aqueous solutions of ammonium sulfate in tap water (concentrations range: 0.25–3.6%wt) were sprayed in order to test the ability to detect and quantify inorganic solutes present only in the condense phase. 3) Five aqueous solutions of ethylene glycol (vapor pressure of 0.06 mmHg at 20 °C) in tap water (0.47–2.38%wt) were sprayed to test the ability to detect and quantify organic solutes that are likely to be present in both gaseous and condensed phases.

### OP-FTIR measurements

Spectral measurements were conducted using RAM2000 G2 OP-FTIR monostatic system (Kassay Field Services Inc.). The OP-FTIR detector gain was set to 50, yielding a voltage range of 8–10 V, depending on the device alignment and the meteorological conditions. Spectra covering the wavenumber range of 500–5000 cm^−1^ where acquired approximately every 4 seconds. Each spraying event included the acquisition of ~120 spectra which were co-added to one spectrum to reduce noise interference. The retroreflector was positioned 30 meters from the sensor and the IR source (i.e., total optical path length was 60 meters).

**Figure 1:**
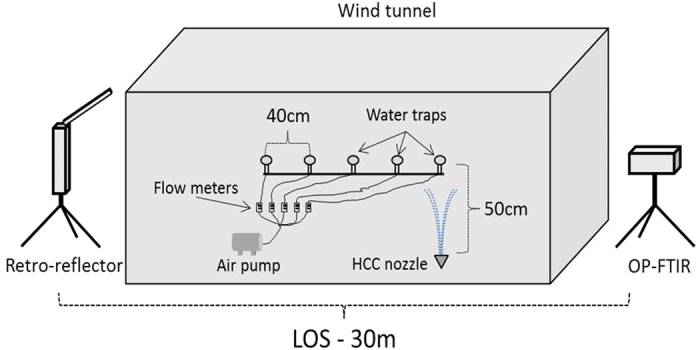
Illustration of the experimental setup inside the spray tunnel.

In order to minimize wind interference the aerosol cloud was generated within a polyethylene tunnel (length: 20 m long, width: 2 m wide, height: 2.5 m) open at both ends (see Fig. 1). A custom system designed to resemble commonly-used agricultural sprayers was used for the dispersion of water based droplets. This system included a centrifugal pump set to 6.5 bars (Pedrollo, 2CPm 160/160) connected to a hollow ceramic cone (HCC) nozzle (ASJ-spray jet). The nozzle was positioned 15 meters from the OP-FTIR and approximately 50 cm below the LOS. The nozzle was directed upwards creating an 80° hollow water cone in the LOS. Five types of HCC nozzles were used (Table 1).

In the first experiment five water loads in LOS were obtained by using a different nozzle in each experiment. In the ammonium sulfate and ethylene glycol experiments (Experiments 2 and 3), only the HCC015 nozzle was used and the different solute loads in LOS were obtained by spraying solutions of different concentrations. Each spraying event lasted approximately 10 minutes with a five-minute interruption between the events. OP-FTIR signals were recorded continuously during and in-between the spraying events.

In addition to the spectral measurements, off-line measurements of water load in the LOS and concentration of the dissolved components in the airborne droplets were conducted. To determine the water load in the LOS, five water traps (glass impingers) where placed at 40 cm intervals up to 1.6 m from nozzle (see Fig. 1). The water traps were positioned at the bottom of the LOS (40 cm above the nozzle) and didn’t interfere with the OP-FTIR measurements. The distance of 1.6 m was set based on preliminary tests which determined that beyond this distance the droplets concentration was negligible. Each water trap was connected to a pump via rotameter (model: VFA-26, Dwyer) withdrawing air at a rate of 40 L/min. The water traps were placed only on one side of the nozzle and symmetric dispersion was assumed. Water concentration *C* (ml_water_/m^3^_air_) near each water trap was calculated by dividing the amount of water collected by the trap by the air flow and the duration of the measurement. Water load (ml_water_/m of optical path) was calculated by integrating the measured water concentration using linear interpolation on both sides of the nozzle, multiplying it by the measurement cross-sectional area and dividing it by the total optical path length:


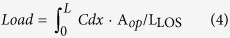


where *Load* is the estimated water load in the LOS, L is the total distance between the water traps, A_op_ is the telescope diameter and L_LOS_ is the length of the LOS.

Ammonium sulfate concentrations were determined by measuring dissolved ammonium using a colorimetric analyzer (nitrogen auto-analyzer, LACHAT Quikchem 8500). Ethylene glycol concentrations were determined using a laboratory FTIR (Bruker Vector 22) with Attenuated Total Reflection (ATR) accessory (Pike Technologies; trough plate ZnSe crystal, 45 degrees). The quantitative analysis was conducted via an ad-hoc calibration curve (peak height vs concentration) created using six standard solutions of ethylene glycol in water (concentration range of 0.2–5%wt). For both salutes, loads in the LOS were estimated by multiplying the calculated water load with the measured concentrations.

Estimation of the amount of water (and ethylene glycol) that evaporated during spraying was obtained from the change in chloride concentration in the spraying solution and the collected droplets. The non-volatile chloride is present naturally in the tap water and by measuring its concentration a mass balance can be computed in order to find the water evaporation percent. The chloride levels were quantified by volumetric titration using AgNO_3_. Mass balance on the basis of water, ethylene glycol and chloride quantities before and after spraying was calculated to retrieve the evaporation percentage.

### Signal analysis and modeling

The signal analysis was carried out using Matlab (V. 8.2.0.701, The MathWorks, Massachusetts, USA). For each spraying event all the spectra recorded during spraying were co-added to reduce noise. Between the spraying events the OP-FTIR measured ambient spectra for five minutes, and the measurements of the last minute were co-added and used as the background signal needed for the extinction calculation (equation (2)). Each extinction signal was further smoothened using zero-phase digital filtering which removes high frequency signals from the spectra. To isolate the spectral signature of the solutes, the spectra recorded during Experiments 2 (ammonium sulfate solution) and 3 (Ethylene glycol solution) were further processed by subtracting from them the clean water signal recorded during Experiment 1 with the same nozzle (HCC015).

For comparison, modeled signals of the different materials (water, ammonium sulfate and ethylene glycol) were calculated using a Mie approximation code originally created by Bohren and Huffman^24^ in Fortran and translated to Matlab. First the extinction cross section was calculated using the Bohren and Huffman procedure^24^ for each particle size and wavelength. The extinction spectrum (left-hand side of equation (5)) was then calculated using the extinction cross section, particles number density and cloud’s length:


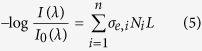


where *σ*_*e,i*_ is the extinction cross section of particle i, N_i_ is the number density of each particle size range (according to size distribution measurements).

When trying to extract a load of any material directly from the spectral measurements we compared the measured signal to the signal calculated via equation (5) (right-hand side). In order to approximate the measured signal to the modeled one, the total number of particles 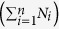 was changed till reaching best agreement between the two. After retrieving the total number of particles, the load was then calculated using equation (6):


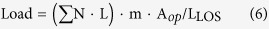


where m is the average mass of the material inside the droplet (or the average mass of the droplets when quantifying water) according to the size distribution.

In Experiments 2 and 3 the condense water signal was subtracted from the measured spectra to cancel the major baseline shift (due to scattering mainly), which interfered with the quantification process. The signals used for subtraction were taken from water-spraying events with the same nozzle type (done in Experiment 1).

**Figure 2:**
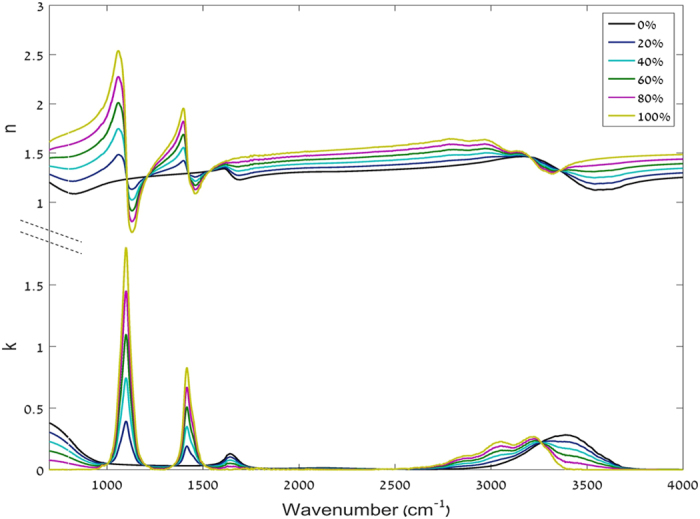
Calculated real (n) and imaginary (k) coefficients of the complex refractive index of aqueous ammonium sulfate.

**Figure 3:**
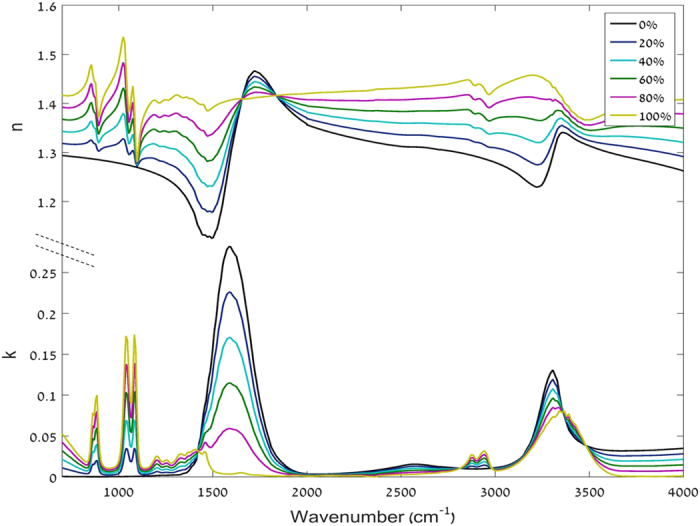
Calculated real (n) and imaginary (k) coefficients of the complex refractive index of aqueous ethylene glycol.

In order to calculate the modeled signals, complex refractive indices of aqueous ammonium sulfate and aqueous ethylene glycol were calculated using the mixing rules for binary solutions (equation (3)) using complex refractive indices of pure ammonium sulfate^25^ and water^26^ and ethylene glycol^27^,^28^ and water^26^, respectively. Figures 2 and 3 show the complex refractive indices of aqueous solutions with concentrations of 0%, 20%, 40%, 60%, 80%, and 100%:

### Size distribution measurements

Complementary measurements with a laser diffraction system (Spraytec, Malvern instruments Ltd.) were carried out to determine the characteristic size distribution of the droplets generated by the spraying system. All five nozzles were tested in a horizontal spraying laboratory setup, in which droplet measurements were done about 1 cm from the nozzle’s outlet. Due to technical limitations only the HCC015 nozzle was tested also in a vertical spraying setup, in which measurements were done 30 cm above the spraying point. The size distribution measured from the vertical experiment was used for modeling signals relevant to Experiments 2 and 3. Each spraying event lasted one minute, the measurement frequency was 100 Hz, and the data was analyzed with the device’s software.

## Results and Discussion

### Experiment 1: Water clouds

The signals acquired during the water spray in Experiment 1 are displayed in Fig. 5. Each signal was obtained when spraying with a different nozzle (resulting in different water loads in LOS). The relevant wavenumber ranges for investigating the water-cloud signals are 800–1300 cm^−1^ and 2000–3000 cm^−1^ (excluding 2300–2400 cm^−1^ due to high CO_2_ absorption). Other wavenumber ranges are excluded due to: low sensor response (below 800 cm^−1^), low IR source illumination (over 3000 cm^−1^) and high water vapor absorption interference (1300–2000 cm^−1^ and over 3000 cm^−1^).

**Figure 4:**
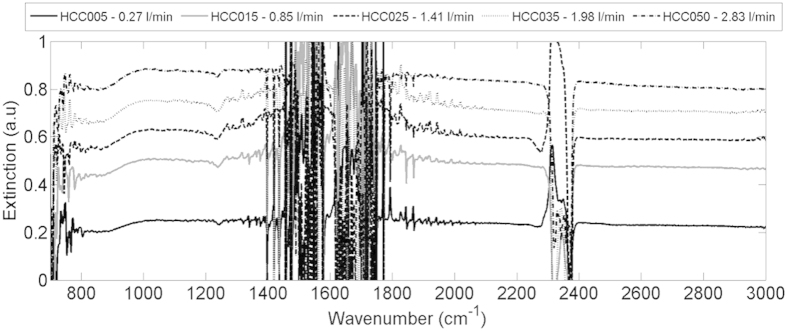
Spectral signals of the sprayed water droplets measured by the OP-FTIR. The intervals of 1300–2000 cm^−1^ and higher than 3000 cm^−1^ were not included in the analysis due to high water vapor interference.

The measured signals appear to be very similar with significant baseline shifts between them (Fig. 4). In the spectral ranges selected for analysis there are very few spectral features resulting from water droplets absorption. In fact, baseline shift is one of the most substantial spectral features created by the water droplets in the Mie scattering range (particle size of 0.1–10000 μm in the mid-IR range). The baseline shift is directly related to the amount of droplets in the LOS (i.e., higher water load in LOS the larger the baseline shift in the IR spectra).

**Figure 5:**
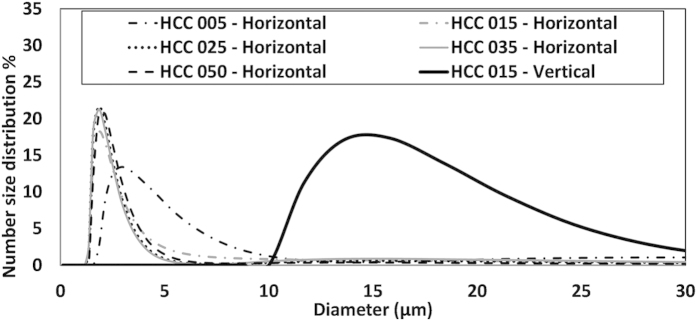
Number size distribution for each nozzle type according to the laboratory laser diffraction (Spraytec) measurements. The vertical size distribution of HCC 015 is the one used for modeling extinction signals.

The very similar spectral features suggest that (despite the different nozzles used) the number size distribution was the same in all these experiments, with the only difference being in the total amount of the water droplets in the LOS. This conclusion is supported by the laser diffraction (Spraytec) measurements, which were conducted in the laboratory to characterize the nozzles output (Fig. 5). These results greatly simplify the data analysis. Different number size distributions would require complicated radiative transfer models for the water load quantification. With the knowledge that the nozzles used have similar number size distributions, the quantification is much simpler and requires only the monitoring of the baseline shift.

**Figure 6:**
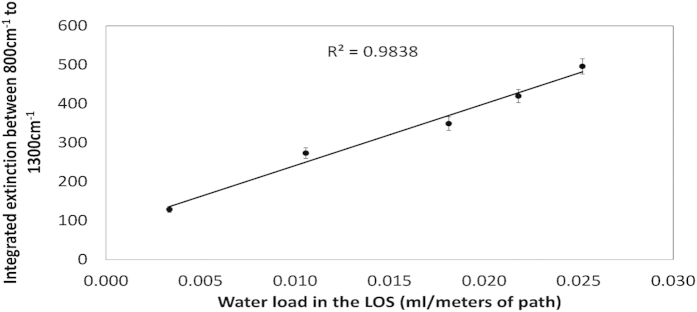
Integrated extinction in the range of 800 cm^−1^ to 1300 cm^−1^ vs. water load in the LOS measured gravimetrically with water traps.

The quantification calibration plot is presented in Fig. 6, showing a linear relationship (R^2^ = 0.984) between the integrated measured extinction of the IR signals (in the wavenumber range of 800–1300 cm^−1^) and the measured water load in the LOS (using the water trap system depicted in Fig. 1). Such linearity supports the previous observation that all tested nozzles have similar number size distributions. The wavelength-dependent extinction cross section of the droplets (σ_e_ in equation (2)) is similar for all nozzles and hence the differences between the extinction spectra are directly related to differences in the total number of droplets (N). From a practical point of view, the calibration curve shown in Fig. 6 would enable rapid quantification of spray clouds generated in similar way with these commonly-used HCC nozzles.

### Experiment 2: Cloud of aqueous ammonium sulfate droplets

**Figure 7:**
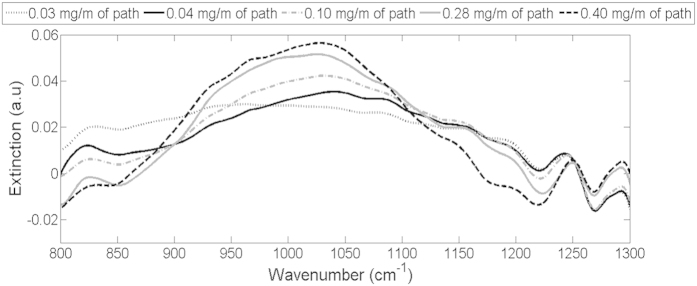
Measured spectra of aqueous ammonium sulfate cloud after subtraction of water signal.

**Figure 8:**
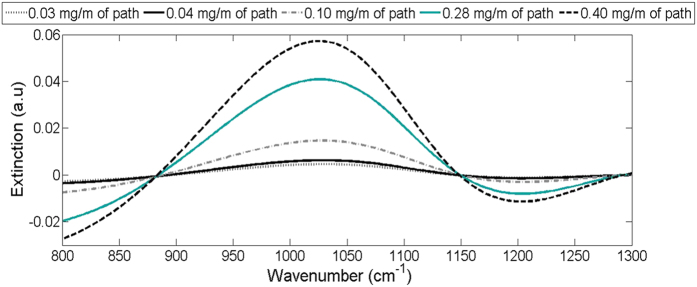
Modeled spectra of aqueous ammonium sulfate cloud after subtraction of water signal.

The goal of the work with ammonium sulfate was to test the possibility to detect the spectral signature of a substance dissolved in the condense phase. Figure 7 shows the measured spectra of the five clouds generated with ammonium sulfate aqueous solutions of different concentrations. The measured extinction spectra presented in Fig. 7 were obtained by subtracting from the average measured signals a similar measurement of tap water in order to isolate the contribution of the ammonium sulfate. The amount of ammonium sulfate in the line of sight, calculated based on ammonium measurements in the water traps and equation (4) (calculated as load of ammonium sulfate per meter of optical path – mg/m), were: 0.03 mg/m, 0.04 mg/m, 0.10 mg/m, 0.28 mg/m, and 0.40 mg/m. For comparison, modeled signals were calculated using equations (5 and 6). First, the total number of particles was obtained from equation (6) using the offline measurements of ammonium sulfate concentration and of the water-load estimated via equation (4). The obtained total number of particles was substitute into equation (5) and the extinction spectrum was calculated, assuming that the droplets’ size distribution in these spray-tunnel experiments was the same as the one measured in the laboratory conditions. The refractive index used for the modeling was calculated using equation (3). Similarly to the analysis of the measured spectra, a modeled spectrum of pure water droplets was subtracted from the modeled spectra of the aqueous ammonium sulfate to isolate the contribution of the ammonium sulfate and the results are presented in Fig. 8.

When comparing the measured (Fig. 7) and modeled (Fig. 8) signals, two main similarities arise: (1) A significant peak between 880–1150 cm^−1^ (with maximal extinction at 1010–1030 cm^−1^) that is positively correlated with (NH_4_)_2_ SO_4_ concentration. (2) Two “crossing points” around 880 cm^−1^ and 1150 cm^−1^. In the measured signals these “crossing points” are not as clearly defined due to interferences. Considering the inherent experimental noise, size distribution fluctuations between measurements and the fact that during the data analysis the spectra were not forced artificially to cross at these points, the similarity between the measured and modeled signal is high.

**Figure 9:**
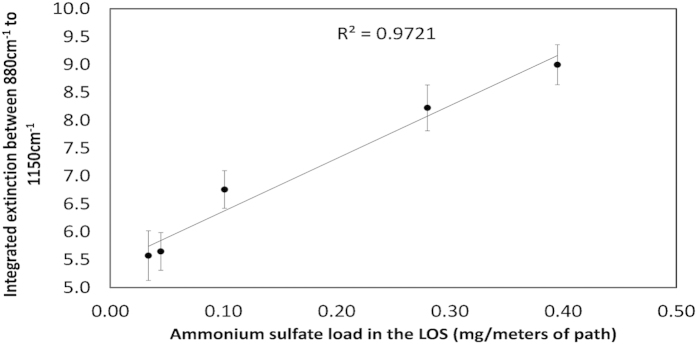
Integrated extinction in the range 880–1150 cm^−1^ as a function of the ammonium sulfate load in the LOS.

Figure 9 demonstrates the linear relationship between the ammonium sulfate load in the LOS and measured signal (integrated extinction in the range of 880–1150 cm^−1^).

Experiment 2 was executed with a relatively constant water load in the LOS as only one nozzle was tested (HCC015). Accordingly, Fig. 9 can be used as-is only to estimate ammonium sulfate load in spraying events with similar water loads. Estimating ammonium sulfate load at different conditions (i.e. different water loads) would require additional correction factors (which were beyond the scope of the present study).

### Experiment 3: Cloud of aqueous ethylene glycol droplets

The detection of aqueous ethylene glycol clouds presents additional challenges since ethylene glycol has a vapor pressure of 0.06 mmHg at 20 °C, and is likely to partition between both phases under ambient conditions. Hence the acquired OP-FTIR signal would be an outcome of the presence of both gaseous and condense ethylene glycol in the LOS.

**Figure 10:**
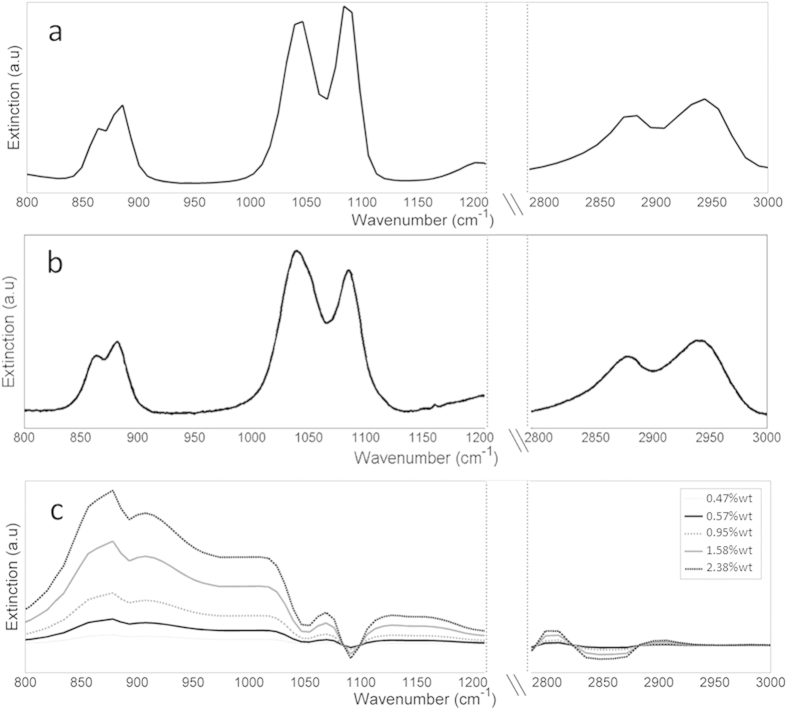
Modeled (**a**) and measured (**b**) spectra of ethylene glycol vapor and modeled spectra of aqueous ethylene glycol droplets (**c**) in different concentrations. Modeled spectra of water droplets with similar size distribution were subtracted from the aqueous ethylene glycol spectra to isolate the ethylene glycol contribution.

Analysis of the ethylene glycol spectra requires knowledge of the spectral signatures of the gaseous ethylene glycol and of the water-ethylene glycol droplets. These spectra were calculated theoretically (Fig. 10a,c), using the refractive indices of ethylene glycol and water, as well as the droplets size distribution measured for HCC015 nozzle. It should be noted that as in the ammonium sulfate spectra, the spectra shown in Fig. 10 were obtained after subtracting the spectral contribution of water droplets with identical size distribution. For comparison, spectrum of gaseous ethylene glycol was also measured using laboratory FTIR (Fig. 10b). All of the modeled and measured spectra are shown excluding regions where the bands are overlapped by water vapor and CO_2_.

The modeled (Fig. 10a) and measured (Fig. 10b) ethylene glycol gas spectra are very similar, showing clear features at the ranges of 850–900 cm^−1^, 1000–1100 cm^−1^, and 2800–3000 cm^−1^. The spectral signatures of the modeled droplets (Fig. 10c) on the other hand differ significantly from the gas signals. The most striking difference is the lack of significant extinction in the 2840–3000 cm^−1^ range for aqueous ethylene glycol droplets. In the range of 800–1150 cm^−1^ the droplets (Fig. 10c) present a strong extinction. The differences between the modeled spectra of droplets and gaseous ethylene glycol is not surprising considering the dominant role of radiation scattering for micron-sized droplets in the mid-IR range.

**Figure 11:**
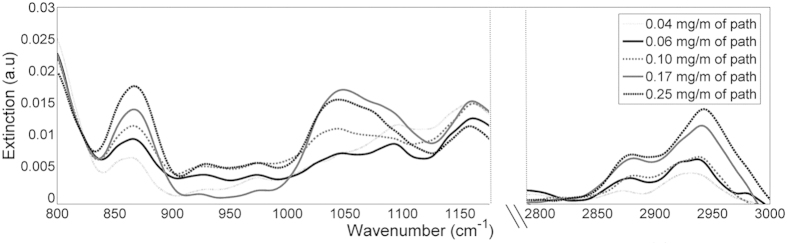
Signals recorded during ethylene glycol spraying experiments. The values in the legend indicates the load of ethylene glycol in the sprayed solution.

The OP-FTIR signals recorded during experiment 3 are shown in Fig. 11. In the range of 2800–3000 cm^−1^, there is a positive correlation between the measured signal and the concentration of the ethylene glycol in the droplets. The fact that this region is associated only with gaseous ethylene glycol indicates that indeed more ethylene glycol in the droplets induces higher evaporation during the spraying event. When comparing the modeled signals to the experimental results in the range of 800–1150 cm^−1^ we can see contribution of the two phases: the measured spectra have two main peaks (like in the gas phase) but with similar heights (the contribution of the condensed phase). Additionally, the shape of the peak around 1050 cm^−1^, which was a doublet in the gas phase spectra has changed to a single peak due to the contribution of the condense phase features. Unlike the previous two experiments (Exp. 1 and Exp. 2), a simple calibration curve cannot be generated directly from the ethylene glycol spectra. Therefore, three alternative approaches were tested to quantify ethylene glycol dissolved in the droplets:

1. Offline quantification using the water traps to collect the droplets and analyzing their ethylene glycol content in the lab.
2. Direct quantification using online OP-FTIR measurements.
3. Indirect quantification using online OP-FTIR measurements.

The direct quantification (#2 above) includes three steps: (a) quantifying gaseous ethylene glycol using inverse modeling applied to the 2800–3000 cm^−1^ interval, where droplets have only very weak spectral signature (Fig. 10c); (b) Subtraction of the gaseous EG (as quantified in the previous step) and of water droplets (using measured signals of water, i.e., signals obtained in Experiment (1) from the measured spectra In the range of 800–1150 cm^−1^; (c) quantifying condense ethylene glycol by comparing the subtracted spectra with modeled signals of water droplets containing ethylene glycol (as shown in Fig. 10c). It should be noted that this last step included only the interferences-free peak centered at ~875 cm^−1^.

Indirect quantification (#3 above) of dissolved ethylene glycol was obtained by calculating the water load (according to the baseline shift calibration curve of Fig. 6) and multiplying it by the concentration of ethylene glycol in the solution used to generate the spray. The main assumption in this method is that ethylene glycol concentration in the droplets is similar to that of the mother solution. Offline measurements (using laboratory FTIR) of ethylene glycol content in collected droplets and sprayed solution confirmed that ethylene glycol concentration stays roughly unchanged (see first two rows in Table 2). Table 2 summarizes the ethylene glycol concentrations obtained via all approaches.

**Figure 12:**
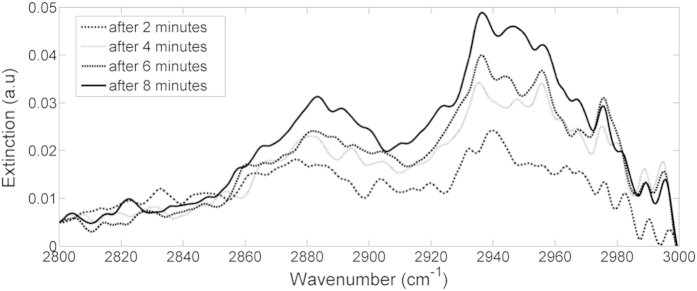
OP-FTIR signal measured during the spraying of ethylene glycol (2.38%wt) using nozzle HCC 015. The spectral features in this spectral range are associated with gaseous ethylene glycol and demonstrate its accumulation over time.

When comparing the results of the gas and condense phase quantification one should bear in mind that there was a major difference between the state of ethylene glycol in both phases: droplets trajectories and deposition resulted in near steady-state conditions for aerosols during experiments (i.e., amount and size of droplets in the LOS is relatively constant), whereas gaseous ethylene glycol accumulated in LOS during the 10 minutes measurement period (Fig. 12).

The root mean square coefficient of variation (RMSCV) measure (equation (7)) was used to quantify the accuracy of the estimates obtained based on the OP-FTIR measurements, considering the offline measurements as ground truth.


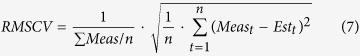


where *Meas* denotes the ethylene glycol’s load according to the offline measurements, *Est* denotes the ethylene glycol’s load calculated from the OP-FTIR measurements, and *n* is the number of measurements. The direct quantification of ethylene glycol in droplets (approach #2) yielded a RMSCV of 57%, and the indirect quantification (approach #3) yielded a RMSCV of 8%. The error of the indirect quantification was clearly much lower but this approach is suitable only for solutes with limited evaporation. Despite the relatively large estimation error reported in the present work, the direct quantification approach may hold great potential for situations in which in assuming low evaporation would not be appropriate. As can be noticed in the description above, this approach requires multiple modeling steps, which should each be improved in future work.

## Conclusions

The goals of the present study were to demonstrate the suitability of active OP-FTIR for quantifying water droplets load in pollutant clouds and to quantify dissolved components inside the droplets. Water load quantification was found to be feasible for the agricultural spraying system used in this study. The fact that the number size distributions generated by the various nozzles was roughly the same allowed a rather simple data analysis based solely on baseline shift. The linear relationship between the signal’s baseline and the droplets’ concentration led to the creation of a calibration curve which could be used to quantify the water load in the LOS. Such simple relationship would no longer be valid if the size distribution was varying, which would require the use of radiation transfer modeling.

Solutes quantification was demonstrated with both non-volatile (ammonium sulfate) and semi-volatile (ethylene glycol) solutes. Measurements of ammonium sulfate content in water droplets in parallel to the spectral measurements demonstrated the capability of the technique to quantify this type of internally mixed components.

The experiment with water droplets containing semi-volatile ethylene glycol presented additional challenges, with the LOS containing this compound in both gas and condense phases. The calculation of ethylene glycol load in the gas phase was relatively simple due to lack of interferences from other compounds in one of the regions of its main spectral signature (2800–3000 cm^−1^). However, the main signature of the condense phase at 800–1150 cm^−1^ was overlapped by signatures of the gaseous ethylene glycol. Direct estimation of the condense phase concentration, by using inverse modeling of the ethylene glycol signatures, resulted in reasonable results only when the region of interest was reduced to 800–900 cm^−1^. More accurate results were obtained indirectly by estimating the water load (from the baseline shift) and multiplying it by the concentration of ethylene glycol in the sprayed solution.

The main limitations of OP-FTIR for quantifying airborne droplets are the dependence of the spectral signatures on size distribution, and the level of noise. Different size distributions alter the signal in a complex and non-linear fashion, causing the extraction of the active ingredient spectral signatures to be dependent on models. Nevertheless, the present work demonstrates that active OP-FTIR can be successfully applied to predict droplets and solutes loads in the LOS.

## Additional Information

**How to cite this article**: Kira, O. *et al.* Detection and quantification of water-based aerosols using active open-path FTIR. *Sci. Rep.* **6**, 25110; doi: 10.1038/srep25110 (2016).

## Figures and Tables

**Table 1:** Nozzles manufacturer (ASJ-spray jet) specifications at operating pressure of 6.5 bars. d_v_50 is the volumetric median diameter.

Nozzle type	d_v_50 (μm)	Flow rate (L/min)
HCC005	~94	0.28
HCC015	~109	0.88
HCC025	~121	1.47
HCC035	~117	2.06
HCC050	~142	2.95

**Table 2:** Ethylene glycol loads on the LOS using different methods of estimation.

	Measured/predicted concentration/load				
	Event 1	Event 2	Event 3	Event 4	Event 5
Sprayed solution	0.47%wt	0.57%wt	0.95%wt	1.58%wt	2.38%wt
Collected droplets	0.48%wt	0.60%wt	1.00%wt	1.65%wt	2.58%wt
Direct estimation- gaseous ethylene glycol	0.097 mg/m	0.142 mg/m	0.158 mg/m	0.307 mg/m	0.404 mg/m
Offline estimation- condense ethylene glycol (app.#1)	0.051 mg/m	0.063 mg/m	0.106 mg/m	0.174 mg/m	0.271 mg/m
Direct estimation- condense ethylene glycol (app.#2)	0.029 mg/m	0.043 mg/m	0.068 mg/m	0.072 mg/m	0.142 mg/m
Indirect estimation- condense ethylene glycol (app.#3)	0.050 mg/m	0.060 mg/m	0.100 mg/m	0.166 mg/m	0.250 mg/m

The ethylene glycol load is given in mg of ethylene glycol per meters of LOS.

## References

[b1] Huang J., Wang T., Wang W., Li Z. & Yan H. Journal of Geophysical Research : Atmospheres. J. Geophys. Res. 119, 398–416 (2014).

[b2] Yan H., Li Z., Huang J., Cribb M. & Liu J. Long-term aerosol-mediated changes in cloud radiative forcing of deep clouds at the top and bottom of the atmosphere over the Southern Great Plains. Atmos. Chem. Phys. 14, 7113–7124 (2014).

[b3] Wang Y., Xia H., Fu J. & Sheng G. Water quality change in reservoirs of Shenzhen, China: detection using LANDSAT/TM data. Sci. Total Environ. 328, 195–206 (2004). 15207584 10.1016/j.scitotenv.2004.02.020

[b4] Somvanshi S., Kunwar P., Singh N. B., Shukla S. P. & Pathak V. Integrated remote sensing and GIS approach for water quality analysis of Gomti river, Uttar Pradesh. Int. J. Environ. Sci. 3, 62–75 (2012).

[b5] Papoutsa C., Hadjimitsis D. G. & Alexakis D. D. Coastal water quality near to desalination project in Cyprus using Earth observation. Proc. SPIE - Int. Soc. Opt. Eng. 8181, 1–8 (2011).

[b6] Richter A. *et al.* Satellite measurements of NO2 from international shipping emissions. Geophys. Res. Lett. 31, 1–4 (2004).

[b7] van Donkelaar A. *et al.* Satellite-based estimates of ground-level fine particulate matter during extreme events: A case study of the Moscow fires in 2010. Atmos. Environ. 45, 6225–6232 (2011).

[b8] Huang J. P., Liu J. J., Chen B. & Nasiri S. L. Detection of anthropogenic dust using CALIPSO lidar measurements. Atmos. Chem. Phys. 15, 11653–11665 (2015).

[b9] Liu J., Chen B. & Huang J. Discrimination and Validation of Clouds and Dust Aerosol Layers over the Sahara Desert with Combined CALIOP and IIR Measurements. J. Metorological Res. 28, 185–198 (2014).

[b10] Lamp T., Weber K., Weidemann J. & Haren G. Van. Application of FTIR Spectroscopy to Open Path Measurements at Industrial Sites in Germany. Proc. SPIE2365, Opt. Sens. Environ. Process Monit. 2365, 6–14 (1994).

[b11] Kasumba J., Holmén B. A., Hiscox A., Wang J. & Miller D. Agricultural PM10 emissions from cotton field disking in Las Cruces, NM. Atmos. Environ. 45, 1668–1674 (2011).

[b12] Moore K. D. *et al.* Particulate-matter emission estimates from agricultural spring-tillage operations using LIDAR and inverse modeling. J. Appl. Remote Sens. 9, 096066 (2015).

[b13] Wu C. F. *et al.* Measurement of fugitive volatile organic compound emissions from a petrochemical tank farm using open-path Fourier transform infrared spectrometry. Atmos. Environ. 82, 335–342 (2014).

[b14] La Spina A. *et al.* New insights into volcanic processes at Stromboli from Cerberus, a remote-controlled open-path FTIR scanner system. J. Volcanol. Geotherm. Res. 249, 66–76 (2013).

[b15] Hashmonay R. A. & Yost M. G. On the Application of Open-Path Fourier Transform Infra-Red Spectroscopy To Measure Aerosols: Observations of Water Droplets. Environ. Sci. Technol. 33, 1141–1144 (1999).

[b16] Wu C.-F., Chen Y.-L., Chen C.-C., Yang T.-T. & Chang P.-E. Applying open-path Fourier transform infrared spectroscopy for measuring aerosols. J. Environ. Sci. Heal. Part A 42, 1131–1140 (2007). 10.1080/10934520701418631 17616885

[b17] Du K. *et al.* Optical remote sensing to quantify fugitive particulate mass emissions from stationary short-term and mobile continuous sources: part I. Method and examples. Environ. Sci. Technol. 45, 658–665 (2011). 21142142 10.1021/es101904q

[b18] Du K. *et al.* Optical remote sensing to quantify fugitive particulate mass emissions from stationary short-term and mobile continuous sources: part II. Field Applications. Environ. Sci. Technol. 45, 666–672 (2011). 21142143 10.1021/es101906v

[b19] Chakrabarty R. K. & Arnott W. P. Journal of Quantitative Spectroscopy & Radiative Transfer Aerosol light absorption and its measurement : A review. J. Quant. Spectrosc. Radiat. Transf. 110, 844–878 (2009).

[b20] Liou K. An Introduction to Atmospheric Radiation. (Academic Press, 2002).

[b21] Lang-yona N., Abo-riziq A., Erlick C., Segre E. & Abo-riziq A. Interaction of internally mixed aerosols with light. Phys. Chem. Chem. Phys. 12, 21–31 (2010). 20024440 10.1039/b913176k

[b22] Heller W. Remarks on refractive index mixture rules. J. Phys. Chem. 69, 1123–1129 (1966).

[b23] Boer G. J., Sokolik I. N. & Martin S. T. Infrared optical constants of aqueous sulfate – nitrate – ammonium multi-component tropospheric aerosols from attenuated total reflectance measurements — Part I : Results and analysis of spectral absorbing features. J. Quant. Spectrosc. Radiat. Transf. 108, 17–38 (2007).

[b24] Bohren C. F. & Huffman D. R. Absorption and Scattering of Light by Small Particles. (Wiley-VCH Verlag GmbH & Co.KGaA, 1983). 10.1002/9783527618156.

[b25] Segal-Rosenheimer M., Dubowski Y. & Linker R. Extraction of optical constants from mid-IR spectra of small aerosol particles. J. Quant. Spectrosc. Radiat. Transf. 110, 415–426 (2009).

[b26] Wieliczka D. M., Weng S. & Querry M. R. Wedge shaped cell for highly absorbent liquids : infrared optical constants of water. Appl. Opt. 28, 1714–1719 (1989). 20548731 10.1364/AO.28.001714

[b27] Sani E. & Dell A. Optical constants of ethylene glycol over an extremely wide spectral range. Opt. Mater. (Amst). 36, 36–41 (2014).

[b28] Sani E. & Dell A. Corrigendum to ‘“ Optical constants of ethylene glycol over an extremely wide spectral range ”’ [Opt. Mater. 37 (2014) 36–41]. *Opt. Mater. (Amst).* **48,** 281 (2015).

